# Home Range Utilisation and Long-Range Movement of Estuarine Crocodiles during the Breeding and Nesting Season

**DOI:** 10.1371/journal.pone.0062127

**Published:** 2013-05-01

**Authors:** Hamish A. Campbell, Ross G. Dwyer, Terri R. Irwin, Craig E. Franklin

**Affiliations:** 1 The School of Biological Sciences, The University of Queensland, Brisbane, Australia; 2 Australia Zoo, Steve Irwin Way, Beerwah, Australia; University of Pretoria, South Africa

## Abstract

The estuarine crocodile (*Crocodylus porosus*) is the apex-predator in waterways and coastlines throughout south-east Asia and Australasia. *C. porosus* pose a potential risk to humans, and management strategies are implemented to control their movement and distribution. Here we used GPS-based telemetry to accurately record geographical location of adult *C. porosus* during the breeding and nesting season. The purpose of the study was to assess how *C. porosus* movement and distribution may be influenced by localised social conditions. During breeding, the females (2.92±0.013 metres total length (TL), mean ± S.E., n = 4) occupied an area<1 km length of river, but to nest they travelled up to 54 km away from the breeding area. All tagged male *C. porosus* sustained high rates of movement (6.49±0.9 km d^−1^; n = 8) during the breeding and nesting period. The orientation of the daily movements differed between individuals revealing two discontinuous behavioural strategies. Five tagged male *C. porosus* (4.17±0.14 m TL) exhibited a ‘site-fidelic’ strategy and moved within well-defined zones around the female home range areas. In contrast, three males (3.81±0.08 m TL) exhibited ‘nomadic’ behaviour where they travelled continually throughout hundreds of kilometres of waterway. We argue that the ‘site-fidelic’ males patrolled territories around the female home ranges to maximise reproductive success, whilst the ‘nomadic’ males were subordinate animals that were forced to range over a far greater area in search of unguarded females. We conclude that *C. porosus* are highly mobile animals existing within a complex social system, and mate/con-specific interactions are likely to have a profound effect upon population density and distribution, and an individual's travel potential. We recommend that impacts on socio-spatial behaviour are considered prior to the implementation of management interventions.

## Introduction

Animals generally confine their movements within discrete areas. The size, placement and shape of the activity space has been termed the home range, and reflects the animals' behavioural repertoire as it searches to procure food, shelter, and mates [1]. For many species social conditions influence the size of the home range, and consequently, the abundance and distribution of the population. Understanding home range dynamics is essential for the pragmatic management of any species [2], but is particularly important in managing top predators because of their influence upon lower trophic levels [3], [4], [5].

The estuarine crocodile (*Crocodylus porosus*) is the apex-predator in its environment and will feed upon a variety of prey items [6]. The species has a wide distribution across northern Australia, occurring in coastal areas, estuaries, rivers, inland swamps, billabongs, and off-shore islands [7], [8], [9]. Unlike a vast majority of the world's apex-predators, the Australian *C. porosus* population has undergone significant growth over the last 30 years. Once in risk of imminent extinction the current Australian population is estimated to be greater than 75 000 non-hatchling individuals [10], [11], [12], [13]. Although the population density varies considerably between river systems [14], such a large growth in population is likely to be altering the dynamics of the wider community and ecosystem [5]. This will occur not only by the consumption of lower trophic animals but also through the alteration of prey species' behavioural ecology [3].

*Crocodylus porosus* are generally considered to be highly territorial animals, with dominant males excluding con-specifics from their home range [15]. More recently however, telemetry studies have recorded large adult male *C. porosus* living in close proximity to each other, thereby refuting previous claims of *C. porosus* as an exclusively territorial species [8], [16]. Understanding which of these social conditions is most apparent is of profound importance towards management because the former would result in social conditions altering population density, dispersal, and distribution whilst the latter would not.

Previous estimates of home range upon *C. porosus* have relied upon either visual sightings or the manual collection of location data via VHF-radio-telemetry [8], [16]. We suggest that the home range estimates of these studies may have been biased by serial autocorrelation because temporal irregularities occurred in the period between location fixing [17]. Furthermore, these studies and others upon crocodilians have defined the home range using mid-stream linear distance or the minimum convex polygon method [18], [19]; whilst these techniques provide a measure of the full extent of the area visited by an individual they ignore patterns of selection within the home range. This is important if we are to assess the difference between an individual's daily usage of an area compared to an area that is merely passed through or only frequented occasionally. In order to make this assessment, kernel utilisation distributions (KUDs) are convenient analytical tools, because they calculate density based upon the entire sample set of relocations during the period of interest rather than the emphasis being on the most outward location points [20]. It was the aim of this study to use KUDs to assess the relationship between daily movements and area utilisation distribution in male and female *C. porosus*. We selected to monitor the crocodiles during the breeding and nesting season (September – February) as the effects of social conditions upon movement and space-use were expected to be most apparent during these periods.

To apply kernel utilisation distribution plots it is important to collect accurate location data at a sufficiently high frequency and regularity [21]. To achieve this, we utilised high precision global positioning system-(GPS) based telemetry data-loggers, which had an inbuilt capacity to parse the collected location data through the ARGOS satellite system. In the light of previous telemetry studies upon *C. porosus* [8], [16], we hypothesised that there would be profound differences in space-use between males and females and the home ranges of individuals would overlap within and between the sexes. Furthermore, due to the high temporal resolution and spatial accuracy of the GPS-based location data, we suspected that new insights into crocodile movement, interaction, and space-use would also be revealed.

## Materials and Methods

### Study site and animals

**Figure 1:**
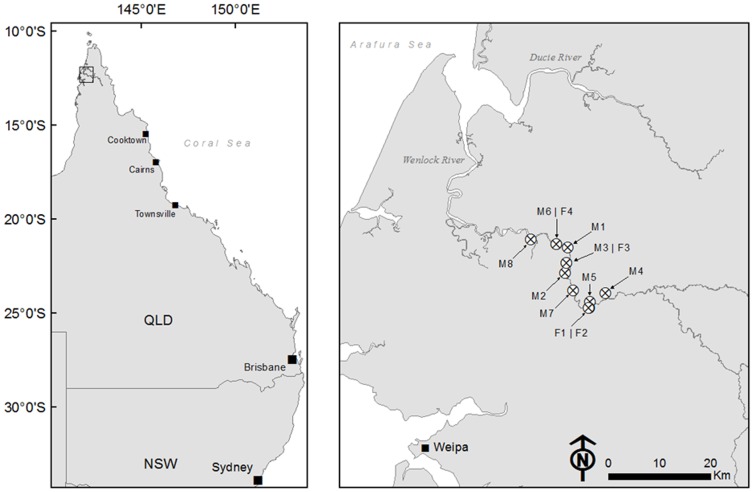
The Wenlock and Ducie River, Cape York, QLD, Australia. The capture locations of each *Crocodylus porosus* tagged for the study are displayed.

Trapping was conducted on the Wenlock River, Cape York Peninsula, Australia during August 2010 (Fig. 1). A field camp was run from the Steve Irwin Wildlife Reserve (142.18°N, −12.38°E). The trapping occurred from the freshwater tidal reaches of the river down to the macro-tidal brackish water, between 20 and 60 km from the river mouth. The bank vegetation in the lower reaches of the trapping zone was mangrove palm (*Nypa fruticans*) changing to *Melaleuca* dominated forests. It has been suggested that out of all the river systems along the western side of Cape York Peninsula the Wenlock system provides the most suitable nesting habitat for estuarine crocodiles [22].

**Figure 2:**
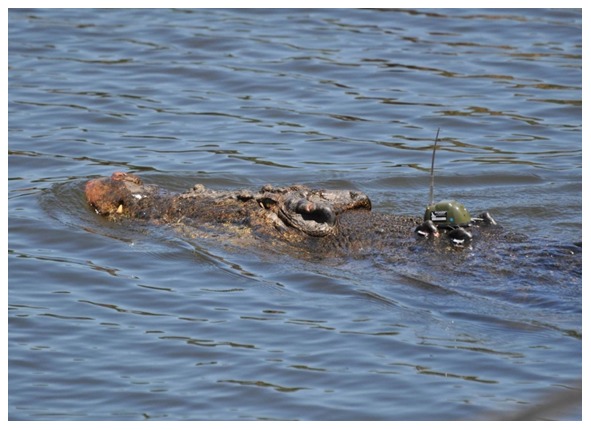
*Crocodylus porosus* with GPS-based satellite transmitter attached to the nuchal rosette.

Adult *Crocodylus porosus* (males = 3.91±0.14 m total length, mean±S.E, n = 8; females = 2.93±0.13 m total length, n = 4) were captured between the non-tidal freshwater reaches of the Wenlock River through to the macro-tidal brackish (Fig. 1). The traps were floated on the water surface or placed at the water edge along the river bank. Each trap was baited with wild pig (*Sus scrofa*) and the trap door was sprung by the crocodile when pressure was applied to the bait, via a trigger mechanism [23]. Once captured, crocodiles were removed from the trap and manually restrained. Total length (TL) and snout-vent length (SVL) measurements were taken and a local anaesthetic (5 ml of Lignocaine, Troy laboratories, Smithfield, Australia) was injected under the nuchal rosette. Once the anaesthesia had taken effect, a single hole was drilled in each of the four raised osteoderms of the nuchal rosette [24]. Stainless steel multi-strand, plastic coated wire (80 kg breaking strain) was inserted through the drilled holes and laced into attachment points on the GPS-based satellite transmitter (in 2009 5×GPS units Sirtrack, Hamilton, New Zealand; in 2010, 13×TGM 410, Telonics, Arizona, U.S.A.). The GPS-units were secured onto the dorsal surface of the crocodile with aluminium crimps threaded onto the stainless steel wire (Fig. 2). The process of removing the crocodile from the trap to eventual release took approximately 60 min. The crocodiles were released at the point of capture. To avoid any bias in crocodile behaviour occurring from the baited traps or increased boat traffic during the trapping period, only GPS-based location data obtained after 01 September were used in the final analysis.

### Data analysis

The devices utilised the global-positioning-system of satellites to determine geographical location twice daily (0800 h and 1800 h). The location data were stored on board the unit and parsed to the ARGOS satellite system between 1000 h–1600 h every other day. For each of the GPS-based location fixes, the accompanying satellite dilution of precision (SDOP) value was used to define the positional resolution and precision. Stationary logging tests (7 d) prior to the study were used to pre-determine the average degree of error for each GPS-unit. All units performed equally and an SDOP of≤3 had a mean accuracy of error 12.1±1.1 m. All location fixes with an SDOP≤3 were excluded from the final analysis.

To assess home range size, we adopted the fixed kernel (FK) method [21]. Kernel density estimators are known to be sensitive to their choice of the smoothing parameter (*h*) [25]. The least-squares cross validation (LSCV) method has been suggested as the most accurate way of estimating the appropriate smoothing parameter [25], it was not however suitable for the present study because it resulted in the delineation of numerous small disjunct contours, excluding connecting stretches of river. A second commonly used smoothing estimator, the reference bandwidth method [26], resulted in large areas beyond the outermost locations being included in the utilisation distributions. To ensure a contiguous home range boundary extending throughout the length of the river and accurately represent the outermost locations, we selected a smoothing parameter of *h* = 750 m. For each individual, the 95% and 50% volume contour of the KUD (hereafter the KUD 95% and KUD 50%, respectively) were determined using the ‘adehabitatHR’ package [27] implemented in the statistical software R [28]. To examine temporal variation in home range use volume contours were constructed for six time periods (01 September–30 September, 01 September–31 October, 01 September–30 November, 01 September–31 December, 01 September–31 January and 01 September–28 February). Crocodile movement was constrained within the river channel, and therefore, the area produced by the FK-method was considered over-representative of the actual area utilised by *C. porosus*. Stretches of river intersecting the volume contours were consequently extracted to ensure that habitat inaccessible to *C. porosus* were not included in the final home range estimates. A high resolution spatial polygon of the Wenlock and Ducie River catchment was constructed using satellite imagery data (Fig. 1) and converted to a 50×50 m raster object using ARCGIS 10 (ESRI, Redlands, California, U.S.A). Areas of river contained within the KUD 95% and KUD 50%, and the corresponding centroid within the KUD 50%, were obtained using functions contained within the ‘sp’ [29], ‘rgdal’ [30] and ‘rgeos’ [31] R packages. This river intersection method reduced the KUD 95% by 90.7±4.1% and the KUD 50% by 71.4±3.2%.

To explore the finer-scale movements in tagged *C. porosus*, two measures of directional movement were investigated. The first measure, the distance moved from the KUD 50% centroid during the period 01 September–30 September, would reveal exploratory movements from the centre of the home range. The second measure, the minimum distance between two locations in series, would reveal periods of activity. As crocodile movements were limited by the trajectory of the river, the minimum distance moved between two locations was calculated along the trajectory of the river using the ‘raster’ [32] and ‘gdistance’ packages [33] in R.

A general linear mixed model (GLMM) was used to assess the influence of body size and sex on movement patterns in *C. porosus*. Daily rate of movement (ROM) was included as the response variable, with days from 01 September (date) and body mass (extrapolated from SVL using the conversion factors in [34]) as covariates, sex as a factor, and crocodile ID as random effect. A second model assessed the relationship between the daily distance each individual was located from the centroid of its KUD 50%, with date and body mass as covariates, sex as a factor, and crocodile ID as random effect. Due to the correlation between body mass and sex the interaction between these variables was included in our model. Analysis was undertaken in Statistica 10 (Statsoft Inc, Tulsa, USA) and P<0.05 was considered significant.

## Results

The majority of the crocodiles tagged in this study remained within the Wenlock River for the duration of the study, but one male travelled to the adjacent Ducie River system, and some males and females moved into seasonal creeks located far upriver. Location data were collected twice daily for eight male and four female *C. porosus* from the 01 September 2010 until the 28 February 2011 (Table 1). 7.1±0.4% of location fixes did not have a sufficiently low SDOP for inclusions in the analysis and were therefore removed from the analysis.

**Table 1:** Summary statistics for four female and eight male *Crocodylus porosus* tracked by GPS-based telemetry between 01 September 2010 and 28 February 2011.

Croc ID	Total Body Length (m)	Day ROM (m/h)	Night ROM (m/h)	Total distance moved (km)	KUD 95% (km^2^)	KUD 50% (km^2^)	Max distance from centroid
**M1**	3.2	301	352	824	34.2*	6.6*	73.2
**M2**	3.7	153	428	1054	42.1*	6.4*	69.2
**M3**	3.9	245	373	1269	72.5*	4.7*	165.4
**M4**	4.3	290	589	1179	9.0	4.2	19.2
**M5**	3.9	67	118	173	7.3	3.5	12.2
**M6**	3.7	84	248	197	8.7	3.6	27.9
**M7**	4.1	166	447	964	11.2	5.1	9.6
**M8**	4.5	200	270	324	7.1	4.2	8.07
**F1**	3.0	123	56	258	12.8	3.9	54.3
**F2**	2.9	17	39	127	7.2	2.2	54.8
**F3**	3.2	34	92	165	4.9	0.8	33.1
**F4**	2.6	27	23	154	1.1	0.5	22.5

* indicates that the monthly kernel utilisation distribution (KUD) had not stabilised by the end of the study.

### Male Movements

**Figure 3:**
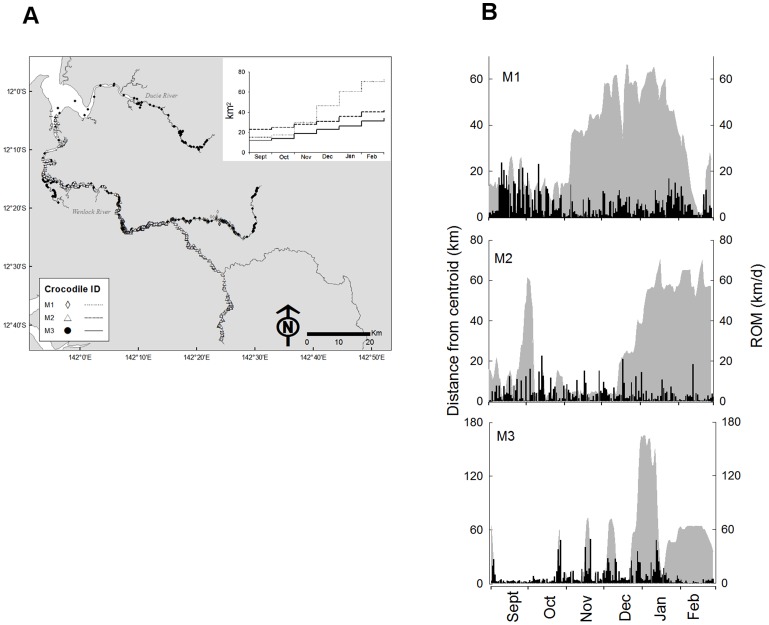
Movement patterns of 'nomadic' male Crocodylus porosus. (a) GPS-location fixes obtained twice daily between 01 September and 28 February (n = 3). Inset line graph shows the monthly cumulative KUD 95% for each individual. (b) The relationship between daily rate of movement (ROM) and daily distance from the KUD 50% centroid (grey  =  primary y-axis; black  =  secondary y-axis).

**Figure 4:**
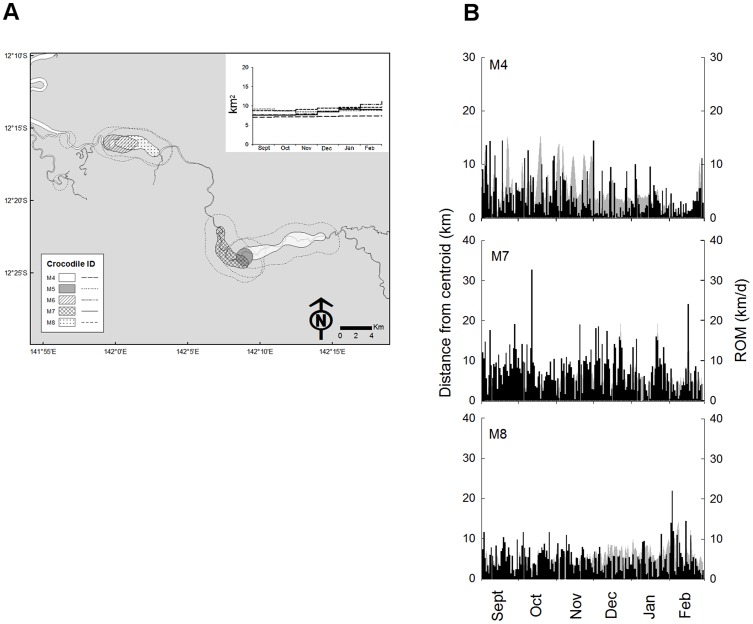
Movement patterns of 'site-fidelic' male Crocodylus porosus. (a) KUD 50% and KUD 95% (dotted boundary) calculated from GPS-location fixes recorded between 01 September and 28 February (n = 5). Inset graph shows the monthly cumulative KUD 95% for each individual. (b) The relationship between daily rate of movement (ROM) and daily distance from the KUD 50% centroid (grey = primary y-axis; black = secondary y-axis).

The application of kernel density estimators to the location data and calculation of the cumulative home range illustrated that the movement patterns of the eight males could be grouped into two discrete categories. The ‘nomadic’ males (n = 3) were defined by the fact they did not demonstrate a stable KUD 95% during the 6-month study (Fig. 3a), whilst ‘site-fidelic’ males (n = 5) displayed a KUD 95% which remained stable throughout the study (Fig. 4a).

The ‘nomadic’ males (n  = 3) travelled extensively throughout the Wenlock and Ducie River catchments (Fig. 3a). They did not confine their movements to a discrete area on the area, and therefore the KUD 50% comprised only a fraction (13.6±4.9%,) of their total KUD 95% (Table 1). Because there was no defined home range, the location fixes rather than the KUDs were plotted on the maps to illustrate space-use (Fig. 3a). The ‘nomadic’ males rate of movement averaged 384.3±29.1 m h^−1^ during darkness and 233.4±56.3 m h^−1^ during daylight hours (Table 1). During the six months of tracking the ‘nomadic’ males moved many hundreds of kilometres and on average travelled 102.6±40.8 km from the KUD 50% centroid (Fig. 3b). The mean total-length of the ‘nomadic’ males was 3.6±0.2 m (mean ± S.E.).

As the name implies the ‘site-fidelic’ males (n = 5) exhibited a stable KUD 50% in which they confined their movements during the 6 months of study (Fig. 4a). The KUD 95% and KUD 50% were comparable across the group (Table 1), and the KUD 50% comprised a large component of the total KUD 95% (48.1±2.8%). There was overlap in the KUD 50% between males, but this was never greater than 47.1% (35.1±6.1% mean±S.E., n = 8). The ‘site-fidelic’ males moved a minimum river distance of 334.4±83.7 m h^−1^ during darkness, decreasing to 161.4±41.2 m h^−1^ during daylight hours (Table 1). Although the average hourly rate of movement for the ‘site-fidelic’ males was less than exhibited by the ‘nomadic’ males, there was no significant difference in the daily distance travelled between the two groups throughout the study (Table 2). The ‘site-fidelic’ males moved back and forward within their home range and therefore the daily distance they were located away from the KUD 50% centroid closely matched the daily rate of movement (Fig. 4b). The maximum river distance the ‘site-fidelic’ males were located away from the KUD 50% centroid averaged 15.4±3.7 km for the group (Table 1). The mean-total length of the site fidelic males was 4.1±0.18 m.

**Table 2:** The results from two general linear mixed-effects models to examine the covariates and factors influencing daily rate of movement (ROM) and site-fidelity for *Crocodylus porosus* (male = 8; female = 4).

		Daily ROM		Daily distance from KUD 50% centroid	
	DF	F	P	F	P
**Sex**	1,9	19.67	0.001	0.99	0.76
**Body mass**	1,9	0.29	0.6	1.3	0.27
**Date**	1,2158	4.8	0.02	629	0.0001

### Female movements

**Figure 5:**
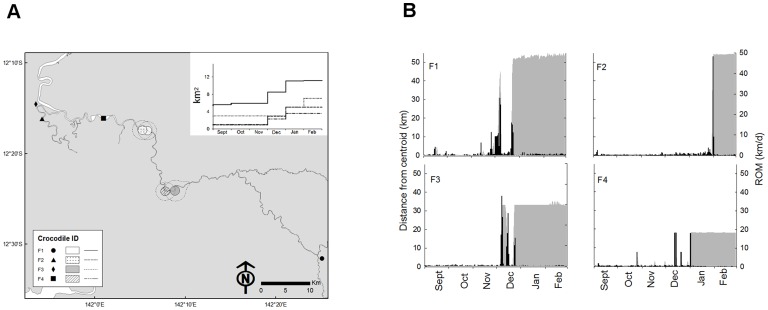
Movement patterns of female Crocodylus porosus. (a) The KUD 50% and KUD 95% (dotted boundary) calculated from GPS-location fixes recorded between 01 September and 28 February (n = 4). Inset graph shows the monthly cumulative KUD 95% for each individual. (b) The relationship between daily rate of movement (ROM) and daily distance from the KUD 50% centroid (grey  =  primary y-axis; black  =  secondary y-axis).

The four tagged female *C. porosus* were of a similar size range and were smaller than the tagged males (Table 1). All females occupied the main trunk of the river and exhibited a defined KUD 95% that was stable between 01 September and 01 December (Fig. 5a). The KUD 50% of two females overlapped at 32.1 and 34.4% area, whilst the other two females held discrete KUD 50% in close proximity. The daily rate of movement for females was much lower than recorded for the males (night = 52.5±13.4 m h^−1^; daylight = 50.3±22.2 m h^−1^), and they did not exhibit the male preference for nocturnal activity (Table 1).

During December and January, each female showed an approximate 30% expansion of their KUD 95%. This increase in the KUD 95% and KUD 50% was due to a sharp increase in daily activity and a lengthening of the distance the female was located away from the KUD 50% centroid (Fig. 5b). F1 travelled upriver whilst F2, F3 and F4 travelled downriver, and within a 24-h period all females were located a considerable distance from the KUD 50% centroid. It seemed logical due to the timing that these long-range movements exhibited by the females were towards nesting areas. F2, F3 and F4 remained at the new location for less than 48 h before travelling back to the KUD 50% centroid within a 24-h period. They remained within their original KUD 50% for 1–2 weeks before undertaking the same journey back to the nesting location. Once at the nesting location for the second time, they remained there until the end of the study (28 February). F1 did not show this repetitive movement and undertook a single long-distance movement in January, remaining at the new location until the end of the study.

### GLMM

The general linear mixed effects model showed that body mass had no significant effect upon the daily rate of movement (ROM) or the river distance an individual was located away from its KUD 50% centroid (Table 2). Sex did have a significant effect upon ROM but not distance from the KUD 50% centroid and date had a significant effect upon both ROM and distance from the KUD 50% centroid. Crocodile ID exerted a significant effect within the model upon both ROM and distance from the KUD 50% centroid, but classifying males into either ‘nomadic’ or ‘site-fidelic’ groups accounted for the significant effect of crocodile ID (F_1,9_ = 67.4, P<0.01).

## Discussion

### Male movements

We recorded two distinct behavioural tactics exhibited by tagged male *C. porosus* throughout the six month study. The daily rate of movement was not significantly different between groups exhibiting either behavioural tactic, but the temporal directionality of movement defined each group. Males exhibiting a ‘nomadic’ tactic ranged throughout the Wenlock and Ducie River catchments; their movement along the river were typically unidirectional upon consecutive days and confined only by river geography. In contrast, males exhibiting a ‘site-fidelic’ tactic confined their movements within a discrete stretch of river. Each individual male maintained its selected behavioural tactic throughout the breeding and nesting season.

The patterns of movement recorded by GPS-based location fixing and defined by KUD home range analysis strongly reflected territorial patrolling behaviour and mate-defence [35], [36]. Tagged con-specifics were located inside the home range of the ‘site-fidelic’ males, but the rate of movement of these individuals would have resulted in them passing through the home range quickly, and the lack of total exclusion may simply be a function of the large home range area and the high mobility of the con-specifics. It is likely that the ‘nomadic’ males passed through the territories of many other untagged ‘site-fidelic’ males during this period.

The present study was undertaken during the breeding and nesting season and all tagged males would have been of reproductive age. Body-size is a good surrogate of social status in *C. porosus* [37], and although behavioural strategy was not significantly segregated by size in this study, we argue that it is the most likely determinate between a ‘nomadic’ or a ‘site-fidelic’ lifestyle. Certainly, the dichotomy of movement patterns were strongly reflective of the ‘fighting’ or ‘sneaking’ alternative reproductive tactics often displayed within polygamous mating systems [38], [39]. That is, dominant males maximise their reproductive success by defending mating rights with co-habiting females, whilst subordinate males maximise their chance by ‘sneaking’ copulations with unguarded females. Further support for this theory in *C. porosus* populations comes from the genetic analysis of eggs collected from nests in the wild, which showed multiple-paternity is widespread with some clutches having more than two sires [40].

A surprising observation that contradicts much of the literature [6], [15] was the sustained high daily rates of movement exhibited by all the tagged *C. porosus*. Even the site-fidelic males travelled hundreds of kilometres during the study, albeit within a discrete area. Translocated male *C. porosus* have been previously reported to have travelled over hundreds of kilometres in a quest to return home [16], [41], [42]. These were however, considered extreme rates of movement, undertaken by the individual only because of the manipulated conditions and a strong homing instinct. On the contrary, high frequency GPS-based location sampling revealed that adult male *C. porosus* are extremely active animals routinely moving many kilometres per day. Presumably, it is because dominant males move back and forth within the confines of a territory that lower rates of location sampling or anecdotal observations have given the impression of far lower potential for movement in *C. porosus*.

### Female movements

In northern Australia, *C. porosus* nest from November through until March [43]. The time between copulation and the laying of eggs in captive *C. porosus* is between 4 to 6 weeks [6], and therefore, courtship and mating may occur anywhere between the end of September and early December. During this period our tagged female *C. porosus* confined their movements within a few kilometres of the main trunk of the river. It has been suggested previously that female *C. porosus* remain close to the nesting location throughout the year [43]. This was not the case in the present study however, and all our tagged females travelled considerable distances (up to 54 km) to a location where we presume they nested (based upon movements that were representative of attentive nest-guarding). Such large movement between the breeding and nesting site has not been reported previously for female *C. porosus*, and may be reflective of the local environment.

The females that were captured and tagged in this study inhabited the tidal freshwater reaches of the river. In this area, the river is relatively narrow and bordered by steep sandy banks sparsely covered with *Melaleuca* trees. The river would be fast-flowing through this section in the wet season, and this location does not contain good nesting habitat for *C. porosus*. Prior to nesting, three out of the four tagged females travelled downstream to a much wider, saline-brackish, section of the river. In this stretch, the river is bordered by thick stands of mangrove, *Nypa* palms and salt-marsh; vegetation and habitat that is much more suited for *C. porosus* nesting [44]. Moreover, this section of the river contains a disproportionally high density of hatchling *C. porosus* compared to other stretches of the river [10]. This suggests that female migration into this area may be a common behavioural strategy within the local population. One of our tagged females did however; migrate over 40 km upstream from the breeding area to the nesting location. This area did not appear to be ideal *C. porosus* nesting habitat [44], but there was a large permanent freshwater swamp in close proximity.

It seems reasonable to assume that the tagged female *C. porosus* travelled long distances to a nesting location because of better nest building materials, access to freshwater, and a reduced likelihood of the nest being flooded during the wet season [44]. What is less clear is why the females did not breed in the locality of the nesting areas and save themselves from these energetically expensive journeys. A possible reason is that the breeding area had better resources than at the nesting areas. Over a four-year period we have laid numerous traps throughout a 60 km stretch of the Wenlock River but only caught females of breeding size within a few discrete locations (Campbell, personal observation). The GPS location data revealed that during the breeding period the females exhibit high site-fidelity to these areas. We argue that these breeding areas are located within productive sections of the river, and the females select these areas in order to build up fat-stores for egg gestation and nesting. If this is true then it suggests that *C. porosus* have a social system based upon resource-based mate choice. That is, the females select areas containing the best resources and the males defend territories around these areas to maximise their mating opportunities [45], [46]. Further investigation is required to confirm this social structure, which would have profound influence upon population density and distribution.

A novel observation of this study was that three out of the four tagged females travelled to the locality of the nest site a few weeks prior to the actual nesting movement. These journeys would have required considerable energetic expenditure, and therefore are likely to have offered some advantage to the offspring. We can only speculate on what this may have been, and the motivation for this repeated-movement so close to nesting remains an avenue of future investigation.

### Effects upon the ecosystem

The movements of the ‘nomadic’ and the ‘site-fidelic’ males would have resulted in very different feeding opportunities and likely required disparate foraging strategies. The ‘nomadic’ *C. porosus* would need to select a variety of prey items from freshwater and saline-brackish ecosystems, whilst ‘site-fidelic’ *C. porosus* would need to take prey whenever it was available within the limits of their home range. Consequently, *C. porosus* are likely to vary in their degree of individual specialisation across spatial scales. Stable isotopic studies upon the tissues of American alligators (*Alligator mississippiensis*) in the Florida Everglades revealed a population composed of both generalist and specialist feeders [46]. There was a strong correlation between ingested prey items and broad-scale movements, and we argue that alternative behavioural tactics driven by social status may have underpinned the observed diet selection by individuals.

When highly mobile predators move rapidly between habitats and feed on a variety of prey species, they create habitat linkages which transport nutrients and energy between systems [5]. A predator that rapidly moves between habitats and switches prey will stabilise the ecosystem by increasing pressure upon one channel of energy whilst freeing up a depleting energy channel from strong predatory pressure [3]. In contrast, a sessile predator may take food whenever available, resulting in negligible transport of energy or nutrients. The dichotomy of movement strategies observed in this study for adult *C. porosus* would result in very different top-down regulation upon trophic interactions and the coupling of ecosystems and habitats. Consequently, understanding the relationship between *C. porosus* density, spatial movement, and home range dynamics are important in defining the wider community and ecosystem effects of a growing *C. porosus* population.

### Implications for management

Since the legislated protection of *C. porosus* there has been a general increase in population abundance across northern Australia. Within some rivers, crocodile density has remained stable for the last 10 to 20 years whilst total crocodile biomass has continued to increase, whereas other rivers are increasing in crocodile density but with no matching increase in total biomass [14]. The social dynamics of the *C. porosus* in this study may aid to explain some of these observed trends. For example, the theory of female resource-based mate choice [47], [48] in *C. porosus* would serve to stabilise population density in areas of good crocodile habitat, and because displacement is unlikely to be achieved by a smaller rival, total crocodile biomass of the area would increase over time. Conversely, rivers or areas with fewer resources would not be selected by females, and dominant males would not hold territories around these areas. Therefore, the population in these poorer quality habitats is primarily composed of smaller subordinate crocodiles, with density but not biomass increasing over time.

Estuarine crocodiles pose a potential risk to the public and a management intervention implemented across northern Australia is to remove crocodiles from around urban centres and areas of high human visitation [48]. A high majority(>75%) of the *C. porosus* captured in permanently set-traps are males between 2 and 3 m total length (Yusuke Fukuda, Scott Sullivan, personal communication), and the high rates of movement exhibited by the subordinate males in this study explains this capture bias. Although implemented less frequently, the removal of dominant male *C. porosus* is also considered as a viable management intervention to reduce crocodile density in particular areas. We recommend that the impact of this management intervention is thoroughly evaluated because, as has been shown for other vertebrate species, dominant male removal can cause social perturbations and can increase movement and immigration from neighbouring areas [49], [50], [51]. Only by thorough evaluation of each management intervention, taking into account any consequences of social perturbation, can the desired outcome be achieved in the management of *C. porosus*.
